# Maximum Expiratory Flow of Children and Adolescents Living at Moderate Altitudes: Proposed Reference Values

**DOI:** 10.3390/healthcare9030264

**Published:** 2021-03-02

**Authors:** Marco Cossio-Bolaños, Rubén Vidal-Espinoza, Luis Felipe Castelli Correia de Campos, Luis Urzua-Alul, José Damián Fuentes-López, Jose Sulla-Torres, Cynthia Lee Andruske, Rossana Gomez-Campos

**Affiliations:** 1 Universidad Católica del Maule, Avenida San Miguel 3605, 3466706 Talca, Chile; 2 Universidad Católica Silva Henriquez, Gral. Jofré 462, 8330225 Santiago, Chile; rvidale@hotmail.com; 3 Universidad del Bio Bio, Avda. Collao 1202 Casilla 5-C, 4050231 Chillán, Chile; lcastelli@ubiobio.cl; 4 Escuela de Kinesiología, Facultad de Salud, Universidad Santo Tomás, 8320000 Santiago, Chile; lurzua@santotomas.cl; 5 Instituto de Investigación en Ciencias de la Educación (IICE), Universidad Nacional del Altiplano de Puno, 21001 Puno, Peru; jovican48@gmail.com; 6 Universidad Nacional de San Agustín de Arequipa, 04000 Arequipa, Peru; josullato@gmail.com; 7 Centro de Investigación Especializada CINEMAROS, 04000 Arequipa, Peru; candruske@gmail.com

**Keywords:** maximum expiratory flow, children, adolescents, percentiles, altitude

## Abstract

(1) Background: Spirometry is useful for diagnosing and monitoring many respiratory diseases. The objectives were: (a) compare maximum expiratory flow (MEF) values with those from international studies, (b) determine if MEF should be evaluated by chronological age and/or maturity, (c) develop reference norms for children, and adolescents. (2) Methods: A cross-sectional study was designed with 3900 subjects ages 6.0 and 17.9 years old. Weight, standing height, sitting height, and MEF were measured. Length of the lower limbs, body mass index (BMI), and age of peak height velocity growth (APHV) were calculated. (3) Results: Values for the curves (p50) for females of all ages from Spain and Italy were higher (92 to 382 (L/min)) than those for females from Arequipa (Peru). Curve values for males from Spain and Italy were greater [70 to 125 (L/min)] than the males studied. MEF values were similar to those of Chilean students ages 6 to 11. However, from 12 to 17 years old, values were lower in males (25 to 55 (L/min)) and in females (23.5 to 90 (L/min)). Correlations between chronological age and MEF in males were from (r = 0.68, R^2^ = 0.39) and in females from (r = 0.46, R^2^ = 0.21). Correlations between maturity (APHV) and MEF for males were from (r = 0.66, R^2^ = 0.44) and for females (r = 0.51, R^2^ = 0.26). Percentiles were calculated for chronological age and APHV. Conclusion: Differences occurred in MEF when compared with other geographical regions of the world. We determined that maturity may be a more effective indicator for analyzing MEF. Reference values were generated using chronological age and maturity.

## 1. Introduction

Pulmonary function may be evaluated by using a variety of simple tests, such as spirometry and maximum expiratory flow (MEF). In addition, more complex and costly tests, such as gas dilution, nitrogen wash, and whole-body plethysmography, may be utilized [1]. In fact, MEF is obtained by means of a maximum forced expiration (in the least amount of time possible) after a maximum inhalation. For this, a simple portable device is required that allows for controlling the capacity to expel air through the bronchial tubes [2] and to register in L/min.

In general, the spirometric values are influenced by genetic, ethnic, and environmental factors. These include nutritional state, altitude of residence, smoking, air pollution, nutrition, socio-economic status, and pulmonary illnesses [3], among other factors. 

Spirometry is useful for diagnosing and monitoring many respiratory diseases. Furthermore, it also has other potential uses, such as estimating the risk of pulmonary cancer, cognitive deterioration, death due to other causes, or death due to cardiovascular diseases [4]. In addition, it allows evaluation of the impact of illnesses on lung functioning related to other organs (heart, kidneys, liver, neuromuscular, or others) [5] as well for assessing respiratory functioning in physical education and therapeutic intervention programs. 

As a result, during the past few years, researchers from various international studies have been interested in developing references for MEF for children and adolescents [6,7,8]. Moreover, some have given priority to proposing equations for predicting MEF values [9,10]. However, to date, no studies have been identified that propose reference values for pediatric populations living in elevated geographic regions.

In fact, inhabitants living at high altitudes are exposed to chronic hypobaric hypoxia where the respiratory system plays a critical role in a series of physiological responses. These allow individuals to adapt and tolerate low levels of oxygen in the tissues at high altitudes [11]. 

In this sense, the proposed MEF references for children and adolescents living at sea level [12], may not be applicable to populations at moderate and high altitudes. This may be due to variations in population during adolescence and accelerated changes in the body shape, proportions, composition, and sexual maturation. Thus, to properly analyze and interpret MEF in children and adolescents living at moderate altitudes in Peru, the researchers proposed to (a) compare the MEF with those of other international studies, (b) determine if the MEF should be evaluated based on chronological age and/or state of maturation, and (c) develop reference norms for children and adolescents living at moderate altitudes in Peru.

## 2. Materials and Methods

### 2.1. Type of Study and Sample

A cross-sectional study was designed with children and adolescents ages 6.0 to 17.9 years old living at a moderate altitude in Peru. The sample size was determined probabilistically (stratified). The sample universe was composed of 28,150 students (13,500 males and 14,650 females) from elementary and secondary schools. Students were selected from 6 public schools in the urban area of Arequipa. The final sample was represented by 13.9% (3909 subjects) (1864 males (6.6%); 2045 females (7.3%)). The number of students selected was proportional to the population based on age range and sex (IC 95%).

Peru is a country with multiple geographic and cultural differences (coastal, mountains, and jungle). For example, the District of Arequipa is comprised of regions with low, moderate, and high altitudes. This study was carried out in the province of Arequipa located at 2320 meters above sea level. During the year, the climate is dry with little precipitation. Temperatures range from 10 °C to 25 °C and with a relative humidity between 46% to 70% [13]. 

### 2.2. Procedures

The anthropometric and MEF evaluations were carried out at installations in each school. A team of four experienced professionals was created to conduct and record the evaluations. These were carried out from Monday to Friday during school hours from 8:30 a.m. to 13:00 p.m. Parents and/or guardians authorized their children to participate in the evaluations by signing informed consent. Students also provided signed consent. 

All students included in the study appeared to be healthy and did not have any illnesses affecting their respiratory system. Parents were asked for the aforementioned information. Students not completing the tests or transferring into the schools from low or high elevations were excluded from the study. All procedures were carried out according to the Declaration of Helsinki for Human Subjects and the recommendations from the Ethics Committee of the Universidad Nacional de San Agustin (UNSA), Arequipa.

Anthropometric evaluations for weight, standing height, and sitting height were carried out based on the recommendations of Ross and Marfell-Jones [14] without shoes, in shorts and a t-shirt. Standing height was measured using a portable stadiometer (Seca & Co. KG, Hamburg, Germany) with a precision of 0.1 mm and a scale of 0–2.50 m. Sitting height was taken with students sitting on a wooden bench (50 cm high) with a measurement scale of 0 to 150 cm with a precision of 1 mm. Body weight (kg) was measured with a digital scale (Tanita, United Kingdom, Ltd.) with a scale of 0–150 kg and with an accuracy of 200 g. Body mass index was calculated using the formula: BMI = Weight/Standing height^2^.

The maximum expiratory flow (MEF L/min) was based on the protocol proposed by Quanjer et al. [15]. A Mini Wright device (Clement Clarke International Ltd., Essex, UK) with a range of 60 to 900 L/min was used to measure the MEF. The evaluation was carried out with students standing without bending the neck. Students were instructed in the techniques of how to perform the maneuvers (a forced inhalation followed by a fast exhalation). Students performed two tries, and of these, the highest was recorded. Prior to the test, students were given 2 to 3 practice tries to familiarize themselves with the evaluation. Quality control of the FEM evaluations was carried out by means of test and retest. The intra- and inter-rater technical measurement error (MTE) was 2 to 3%.

The age of peak height velocity growth (APHV) was calculated with the anthropometric equation proposed by Mirwald et al. [16]. The state of biological maturation can be determined using this equation. This technique indicates the time before or after APHV was calculated by using specific formulas for each sex and variables, such as chronological age, weight, standing height, sitting height, and length of the lower limbs. A positive result indicated a more advanced state of maturation whereas a negative result meant a less advanced state. Zero (0) indicated the child was experiencing PHV (peak height velocity).

Considering that the MEF may vary according to age and sex, especially between geographic regions. To compare the MEF references between geographic regions, percentiles proposed by age and sex from Italy [7], Spain [6], and Chile [8] were used. In all cases, percentile 50 was the reference for comparison. 

### 2.3. Statistics

The Kolmogorov-Smirnov (K-S) test was carried out in order to establish the normality of the distribution of the data by age and sex. Descriptive statistics were calculated of the arithmetic mean, standard deviation, and range. The significant differences between males and females were verified through the t-test for independent samples. The comparisons between the reference studies were graphed (percentile 50). Correlations between variables were carried out by means of the Pearson test. In addition, regression analysis was carried out in steps. The percentage of explanation R^2^ and standard error of estimation (SEE) were calculated. Percentile curves (P3, P5, P10, P15, P25, P50, P75, P85, P90, P95, and P97) were created with the LMS [17] method. The LMS method was adjusted based on three smoothed curves (L(t) Box-Cox Power, M(t) median, and S(t) Co-efficient of variation). The software LMS Chart Maker version 2.3 [18] was used. Statistical calculations were carried out with SPSS 16.0. The level of significance adopted for all cases was 0.05.

## 3. Results

The anthropometric values, APHV (state of maturation), and MEF by age and sex are illustrated in Table 1. No significant differences occurred in weight, height, and MEF for both sexes from age 6 to 11 years old. From 12 years old to age 17, males showed greater weight, height, and MEF (*p* < 0.05). For sitting height, the values were similar from age 6.0 to 14.0 years old. However, from 15 years old to 17, males showed greater sitting height (*p* < 0.05). Significant differences occurred only in BMI for males at age 17 where they showed greater BMI. For the state of maturity (APHV), at early ages (6 to 8 years), no differences were found. When compared to males, the females experienced an advanced state of maturation until age 17 years old (*p* < 0.05).

**Figure 1:**
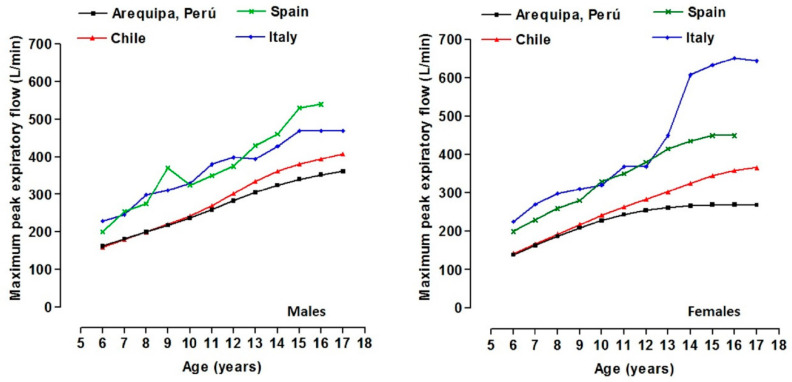
Comparison of the MEF by age and sex with international references for Spain [6], Italy [7], and Chile [8].

The comparisons between the curves of international studies are displayed in Figure 1. At all age ranges, the values of the curves (p50) for Spain and Italy are higher than those reported in Arequipa (Peru). For females, the values for Spain and Italy were higher between 92 and 382 (L/min) and between 70 and 125 (L/min) for males. In relation to the Chilean student sample, the values were similar in both sexes from age 6 to 11 years old. However, from 12 years old to age 17, the MEF for the students from Arequipa (Peru) was relatively lower. Males demonstrated lower values between 25 and 55 (L/min) and females between 23.5 and 90 (L/min).

**Figure 2:**
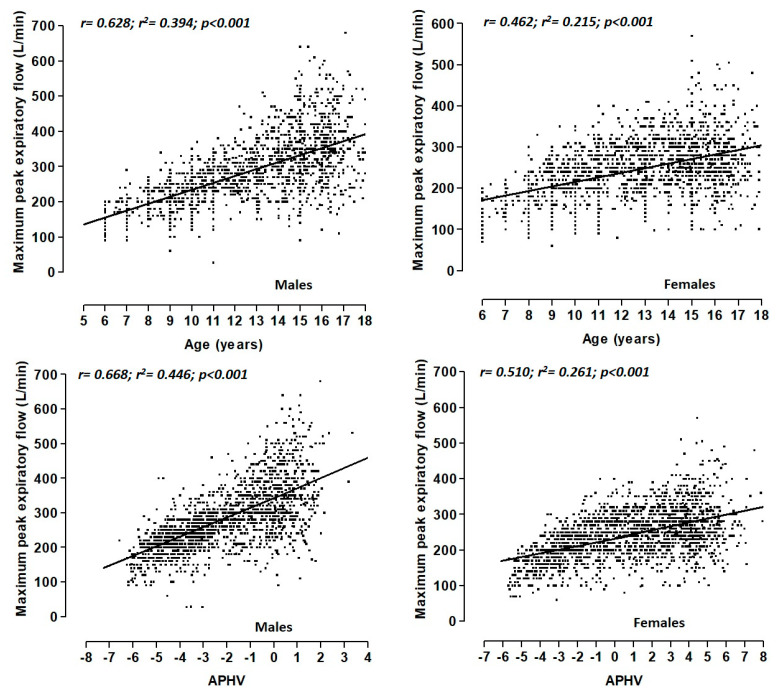
Relationship of the MEF with chronological age (years) and state of maturation (APHV) for both sexes, showing the line of best fit to the point cloud.

The MEF values are arranged by chronological age (years) and maturation (APHV) in Figure 2. Correlations between chronological age and MEF for males was from (r = 0.68, R^2^ = 0.39), and for females, it was from (r = 0.46, R^2^ = 0.21). Correlations for males for the state of maturity (APHV) and MEF were from (r = 0.66, R^2^ = 0.44), and for females, they were from (r = 0.51, R^2^ = 0.26). In all cases, the significant values were (*p* < 0.05).

**Figure 3:**
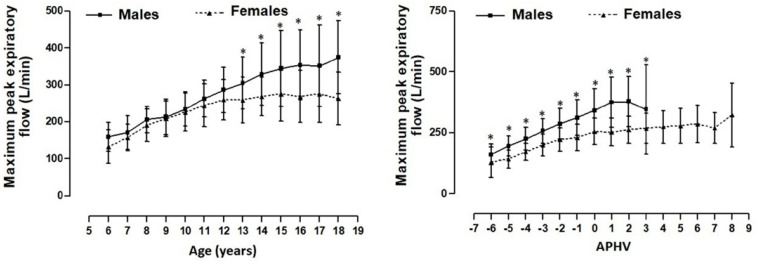
MEF values based on chronological age and maturation (APHV). Legend: * significant difference (*p* < 0.05) related to females.

Figure 3 shows the MEF values for both sexes as well as by chronological age and by maturity (APHV). When these were arranged by chronological age, no differences occurred between ages 6 to 11 years old. However, significant differences (*p* < 0.05) occurred from 12 until 17 years old. When the values were arranged by age of maturation, the results reflected significant differences from 04APHV to 3APHV (*p* < 0.05).

Table 2 depicts the percentiles created with the LMS method (P3, P5, P10, P15, P25, P50, P75, P85, P90, P95, and P97) to evaluate the MEF by chronological age as well as the state of maturity (APHV). In both cases, the values increased with age as APHV advanced.

## 4. Discussion

The findings of this research demonstrate that children and adolescents at moderate altitudes in Peru presented lower values of MEF compared to the studies carried out with Caucasian children from Italy [7] and Spain [6].

The results of this study are corroborated with research carried out with African American [19], Japanese [20], East Indian, Chinese [21], and Chilean [8] children. In these studies, the variability of the MEF was clearly visible between these pediatric populations from different parts of the world.

In general, lung function measurements, unlike many other medical observations, are frequently related to body size and age. Here, height is an indicator of chest size and, and age is an indicator of maturity [9]. 

In the present study, altitude played a key role in the mechanical properties of the respiratory muscles, as the students in this research presented lower body size than their low altitude counterparts in Spain, Italy, and Chile [6,7,8]. Furthermore, other studies focusing on moderate altitudes have shown that these populations tend to present a slight delay in growth and somatic maturation [13]. In fact, the variations in MEF observed in the children and adolescents at moderate altitudes in Peru may be a product of the delay in physical growth that children and adolescents living at moderate and high altitudes tend to present [22]. 

Therefore, it was expected that the children and adolescents from Arequipa would present some delay in physical growth. As a result, this could reflect smaller MEF values than those of their peers from Spain, Italy, and Chile [6,7,8]. Thus, body size plays an important role in the mechanical properties of the respiratory muscles, such as the diaphragm, intercostals, abdominals, and accessories (scalenes, sternocleidomastoids, and intercostals). These interact to produce muscle force during a forced exhalation.

As a result, despite what has been previously described, it cannot be set aside that other factors, such as exposure to environmental contamination, type of occupational activity, and socio-economic status may influence the inter-individual variation in MEF in diverse populations of the world [23]. Thus, future research studies need to include not only populations at sea level but also those at moderate and high altitudes. 

With regard to the analysis of MEF by chronological age and biological maturity, this research demonstrated that when compared by chronological age, the differences became apparent commencing at 12 years old and onwards. 

However, when arranged by state of maturity, differences were present from infancy to adolescence. Furthermore, R^2^ for maturity explained 4 to 6% more than chronological age. This showed that the use of the APHV could be considered as an alternative criterion and acceptable for analyzing the MEF of children and adolescents at a moderate altitude in Peru. 

In some studies, the state of maturation has been reported as the relevant control in samples of children and adolescents [24]. This is especially true when MEF is analyzed on an individual basis in conjunction with the pressure of the manual grip as a possible predictor of bone health [24]. 

In this sense, childhood and adolescence represent an accelerated growth period of lung capacity and forced respiratory flows [25] where the number and size of the alveolar, the form and rigidity of the thorax, and muscle strength undergo significant changes [26]. Although lung development is a continual process during childhood and adolescence, pulmonary functioning depends on age, sex, height, and ethnic origin [27]. In addition, forced expiratory volumes increase significantly during this period between males and females, but a varying rates [28]. This evidently reinforces the idea that the assessment of the MEF in pediatric samples should not only be analyzed by chronological age and height but also based on maturity.

From this perspective, based on the previous results, for this study, percentiles were created. These were used to evaluate MEF based on chronological age and state of maturation (APHV) for children and adolescents living at moderate altitudes in Peru.

These reference values may be useful for evaluating and following respiratory [5], diseases in clinical settings as well as in the field of research. Furthermore, the use and application of these curves are limited to school environments. These cover a wide range of ages that includes elementary (6 to 11 years old) and secondary (12 to 16 years old) students from the Peruvian educational system.

In fact, it is widely known that muscle training improves exercise tolerance, muscle strength, dyspnea, fatigue, and quality of life for individuals [29]. These types of references may be used to classify the deterioration of various physiological systems, such as, for example, cardiac, renal, hepatic, neuromuscular, among others, in addition to evaluating and monitoring functional capacity in physical activity programs and therapeutic interventions. 

The cut-off points adopted for the proposed percentiles were based on a previous study [24], where >p15 was normal, p10 to p15 identified mild inspiratory muscle strength, p5 to p10 was moderate, and <p5 was considered serious. However, the number of categories and exact cut-off points were arbitrary, and, in general, they served to classify the strength of the respiratory muscles [30]. Some other studies highlighted that it is only possible to confirm suspected disorders when the values are found to be below p5 [5,15]. 

The references proposed here based on chronological age may overestimate and underestimate the MEF values. However, when controlling for maturity status (APHV) the MEF values reflected better results in relation to chronological age.

Some limitations of this study need to be pointed out. Due to the nature of the cross-sectional sample, it is not possible to verify the changes during childhood and adolescence. Therefore, the results should be interpreted with caution. Thus, future research projects need to be designed as longitudinal studies. Furthermore, during this research, it was not possible to control for some of the variables, such as students’ physical activity levels and the absence of a control group of students living at sea level. Thus, such information would have assisted in providing a better interpretation of the results obtained in this study. In addition, it is necessary to emphasize that based on the sample selection and size, and the rigorous protocols followed, the results obtained from this research may be generalized to other contexts with similar characteristics to those of the students from this study. 

## 5. Conclusions

In conclusion, the results from this study demonstrated that children and adolescents living at a moderate altitude in Peru presented lower MEF values than their peers from other geographic regions of the world. In addition, the results confirmed that maturity (APHV) may be a better non-invasive indicator for analyzing MEF in relation to chronological age. These inter-individual and geographic variations allow the development of reference values for estimating MEF based on maturation and including by chronological age for both sexes. These results suggest the use and application in physical education programs in educational settings and in public health contexts for diagnosing and monitoring respiratory state in pediatric populations at moderate altitudes. The online calculations for this research may be accessed using the following link: http://reidebihu.net/flujoaqp.php (accessed on 31 January 2021).

## Figures and Tables

**Table 1:** Variables representing the sample studied.

Ages (years)	N	Weight (kg)		Standing Height (cm)		Sitting Height (cm)		BMI		APHV		MEF (L/min)	
		X	SD	X	SD	X	SD	X	SD	X	DE	X	SD
Males													
6.0–6.9	59	26.7	5.5	121.4	5.3	64.8	4.0	18.1	3.2	−5.9	0.3	159.8	38.1
7.0–7.9	85	25.9	5.7	125.3	5.7	66.1	3.5	16.4	3.1	−5.4	0.3	171.7	45.4
8.0–8.9	136	30.4	7.3	129.3	5.5	69.0	3.4	18.1	3.8	−4.8	0.3	206.2	35.9
9.0–9.9	166	34.5	9.0	133.6	6.3	71.2	4.1	19.1	3.7	−4.2 *	0.4	214.1	48.2
10.0–10.9	194	39.5	9.6	139.7	6.9	74.1	4.4	20.1	4.0	−3.5 *	0.4	235.0	45.8
11.0–11.9	176	44.2	10.2	144.3	6.8	75.3	3.6	21.1	4.2	−3.0 *	0.4	263.0	50.0
12.0–12.9	166	44.0	10.1	148.2	8.0	77.1	4.3	19.9	3.6	−2.3 *	0.5	286.8 *	61.1
13.0–13.9	199	49.0	10.7	154.5	8.4	80.4	4.3	20.5	3.8	−1.5 *	0.5	305.5 *	69.8
14.0–14.9	209	55.8 *	12.7	161.3 *	7.7	83.9	4.9	21.4	4.1	−0.6 *	0.7	329.4 *	84.8
15.0–15.9	211	60.5 *	12.8	165.6 *	6.7	85.5 *	4.6	22.0	4.1	0.1 *	0.6	344.2 *	102.3
16.0–16.9	192	59.9 *	11.5	166.9 *	6.1	86.3 *	3.9	21.5	3.7	0.6 *	0.6	354.3 *	94.0
17.0–17.9	71	59.9 *	9.5	168.6 *	5.6	87.0 *	4.9	21.1 *	3.2	1.2 *	0.8	351.5 *	110.5
Total	1864	46.5	15.4	149.4	16.1	78.1	8.0	20.3	4.1	−2.1	2.1	284.3	94.2
Females													
6.0–6.9	60	24.4	4.6	118.7	5.0	62.9	2.9	17.4	3.6	−5.3	0.3	133.2	45.6
7.0–7.9	76	25.7	5.3	124.2	7.0	65.4	4.0	16.7	3.4	−4.5	0.4	158.2	36.1
8.0–8.9	148	30.8	8.0	129.4	7.1	68.8	3.8	18.2	4.0	−3.5	0.5	191.2	43.3
9.0–9.9	179	33.5	9.0	134.5	7.6	71.2	4.3	18.4	3.8	−2.5	0.6	209.0	48.3
10.0–10.9	199	36.6	11.2	139.2	7.2	73.2	4.2	18.7	5.1	−1.5	0.7	226.7	50.7
11.0–11.9	208	44.3	9.8	145.1	6.4	75.5	4.7	21.0	4.3	−0.4	0.7	244.9	58.5
12.0–12.9	189	45.5	10.0	148.3	6.3	78.0	3.4	20.5	3.6	0.8	0.7	260.0	54.3
13.0–13.9	219	49.5	11.0	151.7	6.0	80.6	3.3	21.4	4.0	1.8	0.8	259.1	62.1
14.0–14.9	245	52.5	8.6	154.3	6.0	82.0	3.0	22.1	3.3	3.0	0.7	269.4	52.6
15.0–15.9	235	53.8	9.3	156.6	6.2	83.1	3.5	21.9	3.7	3.9	0.7	275.9	74.4
16.0–16.9	218	53.1	7.8	157.1	5.9	82.9	3.2	21.5	2.9	4.7	0.7	269.4	70.1
17.0–17.9	69	57.1	9.7	157.6	7.1	83.1	5.4	23.0	3.3	5.7	1.0	276.0	77.3
Total	2045	44.6	13.3	146.2	12.6	77.2	7.0	20.5	4.2	0.8	3.1	244.3	68.4

Legend: H: Height; X: Average; SD: Standard deviation; APHV: Age of Peak Velocity Growth; MEF: Maximum Expiratory Flow, (* = 0.005).

**Table 2:** MEF percentiles (L/min) based on chronological age and maturity (APHV) for both sexes.

Group	L	M	S	P5	P10	P15	P50	P85	P90	P95	L	M	S	P5	P10	P15	P50	P85	P90	P95
	Males										Females									
Ages (years)																				
6.0–6.9	1.23	163	0.21	103	117	126	163	197	205	217	1.18	139	0.25	79	93	102	139	175	184	196
7.0–7.9	1.22	182	0.21	117	132	142	182	220	229	242	1.15	164	0.24	96	111	121	164	205	214	228
8.0–8.9	1.20	200	0.20	131	147	157	200	242	251	265	1.13	188	0.24	113	130	141	188	233	244	260
9.0–9.9	1.18	218	0.20	143	160	171	218	263	273	289	1.12	210	0.23	128	146	159	210	259	271	288
10.0–10.9	1.13	237	0.20	156	174	186	237	287	298	315	1.10	229	0.23	141	161	174	229	282	294	313
11.0–11.9	1.05	260	0.21	169	189	203	260	316	329	349	1.05	244	0.23	153	173	187	244	301	314	334
12.0–12.9	0.97	283	0.22	181	204	219	283	348	364	387	0.96	255	0.23	161	182	196	255	315	330	351
13.0–13.9	0.88	306	0.24	191	215	232	306	381	399	426	0.86	262	0.23	166	186	201	262	326	341	364
14.0–14.9	0.82	325	0.25	196	224	243	325	412	433	464	0.75	267	0.24	168	189	203	267	334	351	376
15.0–15.9	0.79	341	0.27	198	228	249	341	438	462	497	0.64	269	0.25	168	189	204	269	342	360	387
16.0–16.9	0.81	352	0.28	196	229	252	352	459	485	524	0.54	270	0.26	166	187	202	270	346	366	395
17.0–17.9	0.85	362	0.30	191	227	252	362	477	505	547	0.45	269	0.27	164	185	200	269	350	371	403
APHV																				
−6	0.36	163	0.26	103	115	124	163	210	223	242	0.79	124	0.24	77	87	94	124	156	163	175
−5	0.51	191	0.24	123	136	146	191	241	254	273	0.92	148	0.23	92	104	113	148	185	193	206
−4	0.62	221	0.23	145	160	171	221	276	289	310	1.03	174	0.23	108	123	133	174	215	224	239
−3	0.71	252	0.22	165	183	196	252	312	327	349	1.12	198	0.22	124	141	152	198	244	254	270
−2	0.77	283	0.23	182	204	218	283	350	367	392	1.18	219	0.22	137	156	168	219	269	280	297
−1	0.81	312	0.24	196	220	237	312	390	409	438	1.20	236	0.22	147	168	181	236	288	300	318
0	0.85	342	0.25	204	234	254	342	433	455	488	1.18	248	0.22	155	177	191	248	303	316	335
1	0.98	369	0.27	205	241	266	369	474	499	535	1.12	256	0.22	161	182	197	256	314	328	348
2	1.24	393	0.29	190	238	269	393	509	535	573	1.02	262	0.23	165	186	201	262	323	338	359
3	1.58	416	0.31	143	220	264	416	540	567	606	0.86	267	0.23	168	190	204	267	332	348	371
4											0.68	271	0.24	171	192	207	271	341	359	385
5											0.48	275	0.25	174	194	209	275	350	370	399
6											0.29	278	0.26	177	196	210	278	359	381	415
7											0.10	280	0.27	179	198	212	281	369	393	432
8											−0.09	283	0.28	181	200	213	283	379	407	452

**Legend**: APHV: Age of Peak Velocity Growth; M: median; L: transformation of Box-Cox; S: coefficient variation; P: Percentile.

## Data Availability

The data is available upon request from the authors.
